# Distinct Network Patterns Emerge from Cartesian and XOR Epistasis Models: A Comparative Network Science Analysis

**DOI:** 10.21203/rs.3.rs-4392123/v1

**Published:** 2024-05-23

**Authors:** Zhendong Sha, Philip J. Freda, Priyanka Bhandary, Attri Ghosh, Nicholas Matsumoto, Jason H. Moore, Ting Hu

**Affiliations:** 1 School of Computing, Queen’s University, 557 Goodwin Hall, 21-25 Union St, Kingston, Ontario, K7L 2N8, Canada; 2 Department of Computational Biomedicine, Cedars-Sinai Medical Center, 700 N. San Vicente Blvd., Pacific Design Center, Suite G540, West Hollywood, CA, 90069, U.S.A.

**Keywords:** epistasis, interaction model, XOR, network science, network analysis, higher-order interactions, community detection

## Abstract

**Background:**

Epistasis, the phenomenon where the effect of one gene (or variant) is masked or modified by one or more other genes, can significantly contribute to the observed phenotypic variance of complex traits. To date, it has been generally assumed that genetic interactions can be detected using a Cartesian, or multiplicative, interaction model commonly utilized in standard regression approaches. However, a recent study investigating epistasis in obesity-related traits in rats and mice has identified potential limitations of the Cartesian model, revealing that it only detects some of the genetic interactions occurring in these systems. By applying an alternative approach, the exclusive-or (XOR) model, the researchers detected a greater number of epistatic interactions and identified more biologically relevant ontological terms associated with the interacting loci. This suggests that the XOR model may provide a more comprehensive understanding of epistasis in these species and phenotypes. To further explore these findings and determine if different interaction models also make up distinct epistatic networks, we leverage network science to provide a more comprehensive view into the genetic interactions underlying BMI in this system.

**Results:**

Our comparative analysis of networks derived from Cartesian and XOR interaction models in rats (*Rattus norvegicus*) uncovers distinct topological characteristics for each model-derived network. Notably, we discover that networks based on the XOR model exhibit an enhanced sensitivity to epistatic interactions. This sensitivity enables the identification of network communities, revealing novel trait-related biological functions through enrichment analysis. Furthermore, we identify triangle network motifs in the XOR epistatic network, suggestive of higher-order epistasis, based on the topology of lower-order epistasis.

**Conclusions:**

These findings highlight the XOR model’s ability to uncover meaningful biological associations as well as higher-order epistasis from lower-order epistatic networks. Additionally, our results demonstrate that network approaches not only enhance epistasis detection capabilities but also provide more nuanced understandings of genetic architectures underlying complex traits. The identification of community structures and motifs within these distinct networks, especially in XOR, points to the potential for network science to aid in the discovery of novel genetic pathways and regulatory networks. Such insights are important for advancing our understanding of phenotype-genotype relationships.

## Background

Epistasis, the interaction between two or more genes, is integral to the study of genetics and is likely ubiquitous in natural systems [1]. However, epistasis is challenging to detect and seldom explored experimentally due to the computational resources required to investigate all possible pairwise and higher-order interactions that can exist between genetic variants [2,3]. Despite this, significant examples of statistical and biological epistasis have been detected in several systems [4–12]. Recently, and of primary interest for this work, results from Batista *et al*. 2023 [12] demonstrate that in two model systems, significant statistical epistatic interactions are not only present, but different interaction models yield distinct results.

Classically, all methodologies that aim to detect epistasis in biological systems use the Cartesian (multiplicative) product of two or more SNPs to model the interaction term. However, this convention is carried over from statistics for ease of calculation. Living systems and genetic pathways are complex and can evolve in various ways to serve a multitude of biological functions [1,13–15]. Indeed, many phenotypes result from large and complex interconnected biological networks [16]. Thus, it is likely limiting to assume that all biological interactions are detectable with a Cartesian product interaction term as it comes with specific model characteristics and assumptions.

In the work of Batista *et al*. 2023, in addition to the standard Cartesian interaction model, the researchers also use the exclusive-or (XOR) penetrance model to investigate epistasis underlying body mass index (BMI) in rats and mice. In the pure and strict XOR model used in this study, the phenotype is dependent only on the multi-locus genotypes (File S1). In other words, the effect of one SNP alone does not provide sufficient information to detect significant associations with the phenotype. Thus, assuming full penetrance and equal allele frequencies in both loci under Hardy-Weinberg equilibrium assumptions, XOR is not linearly separable or detectable using any single-locus analyses like GWAS (File S1; [17]). In their work, the XOR interaction model was selected due to its extreme difference compared to the Cartesian model and its assumed lack of biological plausibility in living systems. Despite this, Batista and colleagues do detect significant statistical epistasis in two model systems, rats and mice, using the XOR model with it yielding more interactions in both species when compared to the Cartesian product model [12]. Furthermore, XOR epistatic loci were significantly enriched for biologically relevant terms and pathways associated with metabolism and BMI, especially in rats. Here, we attempt to better understand the complex associations detected in the rat (*Rattus norvegicus*) system by Batista and colleagues under both interaction models using network analysis.

In light of these intricate genetic interactions, it becomes evident that the complexity of biological systems is largely attributable to the interconnected networks of genetic variants [18], extending beyond the scope of single-gene abnormalities typically seen in Mendelian traits and diseases [19–21]. This complexity underscores the necessity of employing network-based approaches to gain a comprehensive understanding of biological systems. By conceptualizing biological entities, such as genes, proteins, and metabolites, as nodes and representing the interactions between them as edges, network-based approaches offer a holistic perspective on the intricate interplay among these entities. Network based approaches assume that the intricacies of biological systems can be deciphered by analyzing the structures within biological networks. To this end, various biological networks, including protein-protein interaction networks [22–24], metabolic networks [25–28], gene regulatory networks [29,30], and epistatic networks [31], have been proposed to describe the complex processes that drive traits and diseases.

Biological networks possess distinct properties that separate them from random networks, leading to various hypotheses on the mechanisms of biological systems. Firstly, these networks are scale-free [32], characterized by a few highly connected nodes known as hubs. This underpins the hypothesis concerning the role of hub nodes, suggesting that perturbations in these highly connected nodes are more likely to impact the outcome of the system than non-hub nodes [33,34]. Secondly, the high degree of modularity [35] within these networks highlights the division of the network into subgroups of closely interconnected nodes, where nodes within the same module are more likely to interact with each other than with nodes outside the module. This concept aligns with the hypothesis that biological entities, such as genes or proteins, involved in the same biochemical process or disease tend to interact more frequently with each other than other nodes, thus forming a localized cluster or module within the larger network [28,36–38]. Thirdly, the small-world property [39] of biological networks ensures short paths between any pair of nodes, implying that perturbations in a node’s state can influence the activity of many nearby nodes and the network’s overall behavior [40]. Lastly, motifs [41], or subgraphs that occur more frequently than expected, underscore the importance of certain structural patterns in carrying out biological functions, such as several regulatory motifs has been identified [42].

Leveraging Gene Set Enrichment Analysis (GSEA) [43] is a vital approach in examining what each network can elucidate from biological networks. An over-representation analysis (ORA) helps score how likely is it that a gene in the list is truly involved in a biological function or pathway [43,44]. It is based on a predefined set of genes assigned to functions/pathways. The evaluation of statistical significance is commonly done using the hypergeometric test or Fisher’s exact test by comparing the overlap with what would be expected by chance [43]. The intricate analysis of these pathways and ontologies is crucial in unraveling the complex interplay within biological networks. By thoroughly analyzing these networks, we can uncover the multifaceted genetic interactions and regulatory mechanisms that underpin epistasis. This understanding illuminates the dynamic interplay between genes and their pathways and enhances our comprehension of how genetic variants influence phenotypic outcomes in a synergistic or antagonistic manner.

**Figure 1.**
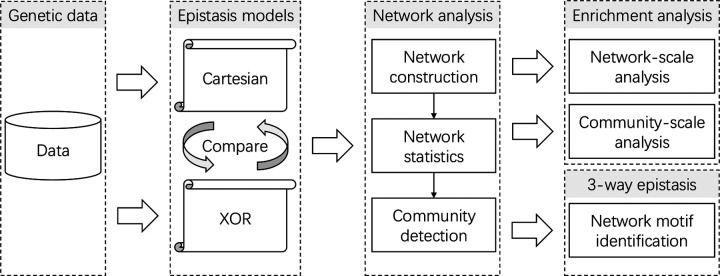
Epistatic network comparative analysis conceptual flowchart: This study evaluates genetic data through two distinct epistasis models to construct and compare epistatic networks. By employing edge thresholding to enhance network modularity, we construct and compare epistatic networks for the analysis of the complex trait of body mass index (BMI) in rats. Our comparative analysis emphasizes the unique insights gained from enrichment analysis at both network and community levels, alongside the identification of network motifs indicative of higher-order epistasis.

In the study conducted by Batista and colleagues, the researchers explored epistasis utilizing two distinct models of interaction: Cartesian (multiplicative) and XOR (Figure 1). Building upon the insights gained from this investigation, our current work employs the principles of network science to analyze whether the networks constructed based on these interaction models exhibit unique structural and topological characteristics. Moreover, this study aims to ascertain whether the specific configurations identified within each network can be correlated with meaningful biological relationships in relation to BMI, obesity, and metabolism in the *R. norvegicus* system. Through this approach, we seek to shed light on the complex genetic underpinnings of a fitness-related trait by deciphering the network-based representations of gene-gene interactions and their potential implications for understanding the genetic architectures of complex phenotypes.

## Materials

### Data source

Genetic and phenotypic data used in this analysis come from an openly available dataset of an outbred, related rat (*Rattus norvegicus*) population, consisting of both males and females, that is derived from eight inbred founders (Heterogenous Stock [45]). Specifically, the genotype and phenotype values we use and used by Batista and colleagues, come from SNPs and phenotype scores utilized in a previously published GWAS [46,47] investigating obesity-related traits in this population of (*R. norvegicus*).

### Epistatic pairs

The methodology of Batista *et al*. 2023 [12] exhaustively tests the interaction terms of every possible pairwise interaction (*n* choose *k*). To test the methodology’s capabilities, Batista and colleagues used 10,000 SNPs with the largest main effects (lowest genome-wide corrected *p*-value) from the (*R. norvegicus*) GWAS [46,47]. The phenotype of interest used in the epistasis analysis was body mass index (BMI) measured from the whole body, including the animal’s tail (BMI TAIL). The methodology allows for the express inclusion of any mathematical operation as the interaction term. Each 10,000 choose 2 combination of SNPs (49,995,000 interactions) were tested for evidence of significant interaction using both Cartesian (multiplicative) and XOR interaction models. Pairs pruned for minor allele frequency and with FDR-corrected *p*-values < 0.05 (Cartesian: 3,438 pairs, XOR: 12,749 pairs) were used in all following analyses.

### Construction of epistatic networks

The construction of an epistatic network involves the identification and selection of epistatic interactions between SNPs in an epistatic pair. As discussed above, all significant pairwise interactions under both interaction models, and the respective SNPs involved in the interactions, from Batista *et al*. 2023 [12] are used to create each, interaction-model specific, epistatic network using the following methods.

### Nodes and edges

In each epistatic network, a SNP is represented by a node. The interactions between these SNPs are represented by edges connecting these nodes. The strength of the interaction is represented by the edge weight, which is typically given by the statistical significance of the interaction (i.e., FDR-adjusted *p*-value).

Not all identified interactions are included in the final epistatic network. A cut-off value τ is determined and only interactions (edges) with their weights (FDR-adjusted *p*-value) less than or equal to this threshold are included. The cut-off value is based on FDR-adjusted *p*-value (edge weight). Once the significant epistatic interactions have been decided based on the cut-off, these interactions are used to construct the network. The resulting epistatic network provides a representation of the most significant epistatic interactions between SNPs.

### Quantifying the extent of community separation

SNPs do not work in isolation but in an interdependent manner to contribute towards phenotypic variation [19,20,31]. Hence, for our epistatic network, we assume the emergence of intensively connected groups of nodes, or communities represents biological functions [20].

Modularity is a measure used in network science to quantify the degree to which the network can be subdivided into clearly separated groups or communities. The modularity (*Q*) is calculated as follows:

(1) $$Q=\frac{1}{2m}\underset{ij}{\sum }\left[A_{ij}-\gamma \frac{k_{i}k_{j}}{2m}\right]\delta \left(c_{i},c_{j}\right).$$

In this equation, m represents the number of edges in the network. The summation term Σ_*ij*_ runs over all pairs of nodes within the network. The adjacency matrix, represented by *A_ij_*, contains elements that are equal to 1 if an edge connects nodes *i* and *j*, and 0 in the absence of a connection. The degree of a node, symbolized by *k_i_* and *k_j_* for nodes *i* and *j* respectively, corresponds to the count of edges connected to the node. The communities of nodes *i* and *j* are indicated by *c_i_* and *c_j_* respectively. The delta function, *δ*(*c_i_, c_j_*), gives a value of 1 if nodes *i* and *j* are within the same community (i.e., *c_i_ = c_j_*), and 0 if they are not. The resolution parameter, denoted by *γ*, establishes a trade-off between intra-group and inter-group edges. In the context of our experiment, the resolution parameter is set to 1.

The value of Q can be either positive or negative with values of high magnitude indicating a strong community structure [48]. If the network has a high modularity (close to 1), this indicates that there is a strong community structure. If the modularity is close to 0 or negative, this indicates that edges are distributed without a clear community structure.

The search for the division of a network into communities that maximizes the modularity score is challenging. This is because there exists an exponential number of The search for the division of a network into communities that maximizes the modularity score is challenging. This is because there exists an exponential number of potential divisions. Therefore, various heuristics and optimization algorithms have been developed to find adequate (though not necessarily optimal) division [49–51]. Based on the size and complexity of the network we studied, we employ the Greedy community detection algorithm [49] for identifying communities within the network. This algorithm utilizes a greedy strategy to identify the community partition that yields the highest modularity. The process of greedy modularity maximization initiates with every node existing in separate communities. It then merges pairs of communities such that a merge results in the maximum increase in modularity. This process is iteratively repeated until the modularity cannot be enhanced further.

### Determination of the epistatic network by tuning edge weight threshold

We introduce an edge weight cutoff τ to select the most significant edges. This threshold determines the edges that are included in the network. The selection of this threshold aims to maximize the modularity of the resulting network.

Formally, we denote the network as *N* = {*V,E,τ*}, where *V* represents the set of nodes (SNPs), *E* represents the set of edges (significant epistatic interactions), and *τ* represents the edge weight threshold. Each edge *e* ∈ *E* that links two nodes *v*_1_*,v*_2_ ∈ *V* has an associated weight, denoted as *ω*(*e*). An edge *e* is included in the network if *ω*(*e*) ≤ *τ*.

To optimize network modularity, we gradually increase the edge weight threshold *τ* and monitor the evolution of modularity. We begin with *τ* set to 0. We then increase *τ* in increments of 0.0001. After each increase, we calculate the modularity *Q* of the resulting connected nodes along with other network metrics, such as the number of connected nodes, the number of edges, and the number of network-connected components. In order to gain a comprehensive understanding of the network’s evolution, we also monitor the number of undirected triangle network motifs. Network motifs are the basic building blocks of the network [52].

We select the *τ* value that yields the highest network modularity as the optimal edge weight threshold. This process ensures that the constructed network *N* not only captures the strongest genetic interactions but also exhibits a community structure that facilitates the identification of functionally relevant modules of SNPs.

### Network comparison

We compare networks generated from XOR and Cartesian interaction models to identify common SNPs, epistatic interactions, and network communities. For the network with the highest network modularity, we perform the comparison at two different scales.

At the scale of nodes and edges of the epistatic network, we consider adjacent SNPs as identical due to linkage disequilibrium (LD). A SNP *n* is represented using a tuple *n* = (*c, p*), where *c* stands for chromosome number and *p* stands for chromosome position. If the difference between a pair of SNPs is within a certain range *δ* and they are on the same chromosome, they are deemed identical. The way we determine identical SNPs can be formally expressed as follows. Given *n*_1_ = (*c*_1_*, p*_1_) and *n*_2_ = (*c*_2_*, p*_2_), we say *n*_1_ and *n*_2_ are the same (within the range *δ*) if:

(2) $$n_{1}\approx n_{2}\Leftrightarrow \left(c_{1}=c_{2}\right)\wedge \left(\left|p_{1}-p_{2}\right|<\delta \right)$$

As for edge comparison, two interactions are considered identical if their SNPs are determined as the same. We represent an edge as a set of two SNPs *e* = (*n*_1_*, n*_2_). Given two edges, $e_{a}=\left(n_{1}^{a},n_{2}^{a}\right)$ and $e_{b}=\left(n_{1}^{b},n_{2}^{b}\right)$. Edges *e*_*a*_ and *e*_*b*_ are considered identical if:

(3) $$e_{a}\approx e_{b}\Leftrightarrow \left(n_{1}^{a}\approx n_{1}^{b}\wedge n_{2}^{a}\approx n_{2}^{b}\right)\vee \left(n_{1}^{a}\approx n_{2}^{b}\wedge n_{2}^{a}\approx n_{1}^{b}\right)$$

As the the range increase from 0 million bases (Mb) to 10 Mb, the number of common SNPs and epistatic interactions will increase.

At the scale of network community, we identify the quantity of common nodes $\Lambda_{\left(C_{a}\to C_{b}\right)}(\delta )$ from community *C*_*a*_ to community *C*_*b*_ with respect to the range parameter *δ*. Let *C*_*a*_ and *C*_*b*_ be two network communities from two different epistatic networks. Each community is a set of nodes, i.e., $C_{a}=\left\{n_{1}^{a},n_{2}^{a},\ldots \right\}$ and $C_{b}=\left\{n_{1}^{b},n_{2}^{b},\ldots \right\}$. A node in the community $n_{i}^{a}\in C_{a}$ will be considered to have a common node in the other community *C*_*b*_ if there exists at least one node that can be determined as similar. The number of such nodes in *C*_*a*_ is used to quantify the similarity from *C*_*a*_ to *C*_*b*_. This similarity is directional, and its formal definition is as follows.

To determine if two nodes *n*_1_ and *n*_2_ is similar, we first define a function *f*_*δ*_ as:

(4) $$f_{\delta }\left(n_{1},n_{2}\right)=\{\begin{array}{cc} 1 & \text{if}\;n_{1}\approx n_{2}\\ 0 & \text{otherwise} \end{array}$$

Using the function *f*_*δ*_, we can define $\Lambda_{\left(C_{a}\to C_{b}\right)}(\delta )$ as:

(5) $$\Lambda_{\left(C_{a}\to C_{b}\right)}(\delta )=\underset{n_{i}^{a}\in C_{a}}{\sum }\underset{n_{j}^{b}\in C_{b}}{\max}f_{\delta }\left(n_{i}^{a},n_{j}^{b}\right)$$

We utilize $\Lambda_{\left(C_{a}\to C_{b}\right)}(\delta )$ to compare each pair of network communities from the two epistatic networks with different interaction models. The comparison of nodes will also consider the range of their position. The number of common SNPs in *C*_*a*_ will evolve as the change of the range parameter *δ*.

We utilize the area under the curve (AUC) to quantify the similarity of community pair as a function of range $\Lambda_{\left(C_{a}\to C_{b}\right)}(\delta )$. To compute the AUC, consider *δ* to have discrete values ranging from a minimum value *δ*_min_ to a maximum value *δ*_max_ with a step size *s*. The AUC can be approximated using the function shown as below:

(6) $$\text{AUC}=\overset{M}{\underset{k=0,s}{\sum }}\frac{\Lambda_{\left(C_{a}\to C_{b}\right)}\left(\delta_{\min}+ks\right)+\Lambda_{\left(C_{a}\to C_{b}\right)}\left(\delta_{\min}+(k+1)s\right)}{2\times (M+1)},$$

where $M=\frac{\delta_{\max}-\delta_{\min}}{s}-1$ represents the total number of steps between *δ*_min_ and *δ*_max_. To reflect the relative size between the number of common nodes and the overall number of nodes in community *C*_*a*_, symbolized as |*C*_*a*_|, the normalized AUC is denoted as:

(7) $${\text{AUC}}_{\text{norm}}=\overset{M}{\underset{k=0,s}{\sum }}\frac{\Lambda_{\left(C_{a}\to C_{b}\right)}\left(\delta_{\min}+ks\right)+\Lambda_{\left(C_{a}\to C_{b}\right)}\left(\delta_{\min}+(k+1)s\right)}{2\times (M+1)\times \left|C_{a}\right|}.$$

In our study, *δ* ranges from *δ*_min_ = 0 Mb to *δ*_max_ = 10 Mb with a step size of *s* = 1 Mb.

### Functional enrichment analysis

#### g:Profiler

Enrichment analysis discerns biological insights from a list of gene names by detecting statistically significant representation of biological functions, such as GO terms and pathways. In our study, we utilize the rat genome (Rnor version 6.0) as the reference genome. We perform our enrichment analysis using g:Profiler [44]. Our enrichment analysis starts with a list of SNPs, each identified by a unique ID consisting of chromosome number and chromosomal position in base pairs (for example 1:12345678). The list of SNPs are from the connected nodes of the epistatic network with the highest network modularity. For every SNP in the list, we include gene models 1 million base pairs upstream and downstream from the original position.

The prepared ranges (for example 1:11345678:13345678) are then directly used as inputs for g:Profiler. Our enrichment analysis is performed using g:Profiler’s R package to query the biological terms related to these ranges. We use the default configuration for each query (supplementary file S1-IV). All available data sources are considered for the analysis to provide a comprehensive understanding of potential biological implications. The biological terms returned by g:Profiler are filtered based on their g:SCS threshold. Only terms with a value less than 0.05 are retained.

The epistatic networks in this work are optimized for modularity to highlight distinct communities of interacting SNPs. To discern potential biological signals, we conduct enrichment analysis at two levels: the entire network scale and the community scale. For the network scale, all SNPs in the network are taken into account for enrichment analysis based on g:Profiler. Conversely, the community scale analysis focuses on each discrete community within the epistatic network. Given that the number of SNPs in each community is significantly lower than in the entire network, the enrichment analysis at this scale yields more targeted functional terms. This specificity provides a contrast to the broader perspective obtained at the network scale.

#### Comparative analysis of g:Profiler biological terms

To acquire a deeper understanding of the enriched biological terms for each interaction model, a comparative analysis is conducted. The table obtained from g:Profiler is organized according to the term sources - GO:Biological Process (GO:BP), GO:Cellular Component (GO:CC), GO:Molecular Function (GO:MF), REAC, KEGG pathways, miRNA and Transcription Factors (TFs) (See Supplementary file S2). Venn diagrams are generated using the terms from both interaction models for each term source (using a custom R script). The parent hierarchy information for each term is extracted using the R package “GOfuncR”. (supplementary file S4)

In addition to analyzing enrichment at the lowest level of the ontological hierarchy, we wished to gain a broader perspective and facilitate the identification of biological differences between Cartesian and XOR networks. Thus, another layer of information is necessary. To achieve this, we focus on the first child of the parent classification for each term. For instance, in the case of GO:BP, the parent is Biological Process, and the first child of interest is the term when going down one level in the GO hierarchy. This selection is made to ensure an adequate level of variability can be captured, as the parent term alone offers no differentiation. In the case of miRNA and TFs, where a hierarchical structure is absent, this analysis is not conducted. Hence, this analysis is only performed for GO:BP, GO:CC, GO:MF, KEGG and REAC. There are extremely few child terms for GO:CC and GO:MF, therefore not much variability can be recorded at this level. Going down one level lower for both of these sources helps capture more variation. Therefore the second-order child is taken into consideration for these terms. Venn diagrams are generated for the five sources.

#### EnrichmentMap

Performing an enrichment analysis on a set of SNPs often yields a large volume of terms, which poses a significant challenge in interpreting the results. Thus, a proper summary of these terms is needed to simplify the process of interpreting and understanding the biological implications of our findings.

EnrichmentMap [53] visualizes functional terms as a network following the principle that terms sharing many genes suggest a higher degree of functional relatedness. In the generated network, each node represents a functional term, and the edges connecting them represent the overlap of genes associated with those terms. Nodes are sized relative to the total number of associated genes, and edges are weighted by the number of shared genes between the two connected terms. EnrichmentMap provides options to control the sparsity of edges in the network, and we configure this option to be sparse. The resulting clusters of related terms provide insight into the themes emerging from the enrichment analysis. EnrichmentMap can merge the results from different queries. In our analysis, node colors represent terms from different epistatic networks of varying interaction models and network communities.

AutoAnnotate [54], a Cytoscape plugin, is employed to further refine the output of EnrichmentMap. This plugin uses WordCloud [55] to generate a label for each cluster of nodes, serving as a theme for that community of terms. The use of AutoAnnotate enables a more intuitive interpretation of the clusters, facilitating our understanding of the functional enrichment results.

## Results

This section presents a comprehensive analysis in three perspectives: Firstly, it explores the unique network structures formed by the XOR and Cartesian interaction models, utilizing edge weight thresholding to optimize network modularity and examine various network metrics. Secondly, it compares these networks, focusing on their similarities and differences in terms of nodes, edges, community structures, and their implications on identifying higher-order epistasis based on lower-order interactions. Lastly, the section delves into enrichment analyses, shedding light on the functional term and biological implications of the networks derived from each interaction model.

### XOR and Cartesian interaction models yield distinct network structures

Edge weight thresholding is employed to optimize network modularity. We also monitor several key metrics during this process: the number of connected nodes, the number of edges, the number of network components, the size of the largest network component, and the number of triangles in the network.

**Figure 2.**
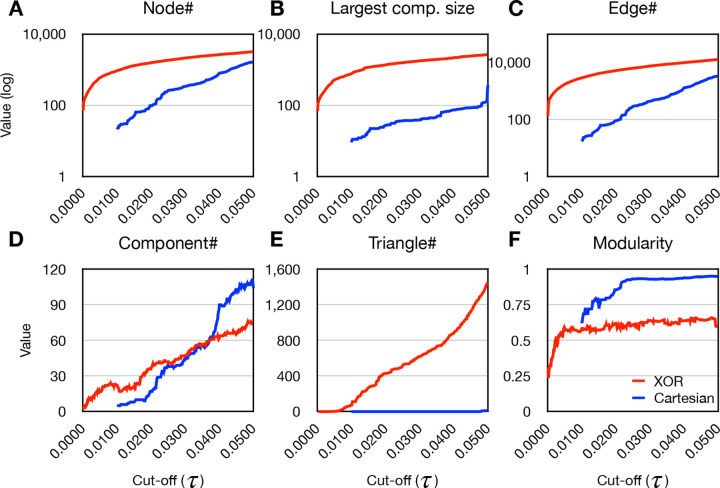
The evolution of network metrics for Cartesian and XOR interaction models. **A-F**, These illustrative figures portray the evolution of network metrics in relation to the edge weight cut-off (*τ*) within epistatic networks employing either Cartesian (blue) or XOR (red) interaction models.

The evolution of network metrics with respect to edge weight cutoff *τ* in the XOR-based epistatic network is shown in Figure 2. This network is dominated by its largest connected component, where the ratio of nodes in the largest component to the total number of connected nodes consistently surpasses 0.8. As *τ* elevates beyond 0.0035, the network experiences the emergence of triangles. At this point, there are 440 connected nodes with 1,295 edges. A maximum modularity metric of ~ 0.667 is achieved when *τ* reaches 0.0434.

The evolution of network metrics in the Cartesian-based epistatic network manifests differently than the XOR model. In this network, the largest connected component does not dominate the network. This is evident as the ratio of the number of nodes in the largest component to the total number of connected nodes is always below 0.5. Another distinctive difference of the Cartesian network is its sparse occurrence of triangles (Figure 2-E). Triangles begin to emerge when the edge weight cutoff surpasses 0.048, with 1,496 connected nodes and 2,852 edges. Even though the Cartesian network does develop triangles, the quantity is much lower than the XOR network, underlining the unique network structures of each interaction model. The Cartesian network reaches a maximum modularity of ~ 0.951 at a cutoff value of 0.0488.

We employ the cut-off corresponding to the highest modularity edge threshold. We also explored the possibility of using the elbow point as the edge threshold (refer to Supplementary File S1-V). However, we ultimately opted not to utilize the elbow point in order to maximize the inclusion of as many SNPs as possible within the network.

### XOR and Cartesian interaction models share similar SNPs but have distinct epistatic interactions

In addition to the metrics at the network scale, this section compares the node set, edge set, and network community allocation of the two epistatic networks with the highest network modularity (Section).

### Comparative analysis of node and edge overlap in XOR and Cartesian interaction models

This section explores the impact of the range parameter on the similarity assessment of nodes and edges in two distinct network models, focusing on how varying the positional range influences the identification of identical SNPs and their interactions.

As elucidated in Table 1, when comparing SNPs with their exact chromosome position (range equals to 0 Mb), the two interaction models exhibit an overlap of 416 SNPs. The comparison for edges, illustrated in Table 2, elaborates that the two networks share 99 common edges (range equals to 0 Mb), encompassing 51 SNPs. The majority of the SNPs connected by these common edges are situated within the chromosomal range of chr1.280924773 to chr1.282730801 which includes the putative quantitative trait locus (QTL) with the largest main effect for BMI TAIL from the original GWAS (chr1.281788173) [46,47].

Further comparative investigation for the two epistatic networks considers the range. parameter *δ* of chromosomal position. The results in Table 1 and Table 2 illustrate that enlarging the base pair range *δ* is associated with an increase in identical SNPs and edges between XOR and Cartesian. Specifically, as the range extends from 0 to 10 Mb, 7.82% (or 840 epistatic interactions) of the interactions in the XOR model can be found in the Cartesian model. On the other direction, the fraction of identical edges in the Cartesian model is 73.35% (or 2,312 epistatic interactions), indicating that the majority of interactions in Cartesian are contained in the neighboring positions of the interactions in XOR (Table 2). Node comparison implies that the SNPs from both models are located close to each other. Table 1 demonstrates that for both models, an increase in range from 0 to 10 Mb allows for the detection of most SNPs in the other model (XOR in Cartesian: 94.76%, Cartesian in XOR: 97.91%). Taken together, XOR distinguishes more epistatic interactions compared to Cartesian, but these additional interactions are situated close to the SNPs in Cartesian.

### XOR exhibits more triangle network motifs than Cartesian

We also observe a remarkable distinction between the two epistatic networks in the number of triangular network motifs. In our comparison, the XOR model (*τ* = 0.0434) is observed to generate 1,033 triangles, which is significantly higher than the nine triangles formed within the Cartesian model (*τ* = 0.0488). This considerable discrepancy suggests that the XOR model potentially captures more complex interactions among nodes within the network.

Another characteristic of the triangles in XOR lies in their chromosomal positions. This may suggest the XOR model has a greater potential for 3-way interactions. We observe that the SNPs of most triangles in XOR are located on different chromosomes. Whereas, the triangles in the Cartesian network are found to be on the same chromosome in close proximity, specifically within the chromosomal range of chr1.280924773 to chr1.282527574. This range overlaps strongly with the position of the SNPs linked by the common edges (with Range equals to 0 Mb) between the two epistatic networks (chr1.280924773 to chr1.282730801). These closely located triangles could be attributed to cis-regulatory epistatic interactions in association with the putative univariate QTL at chr1.281788173 [46,47], or false positives.

**Figure 4.**
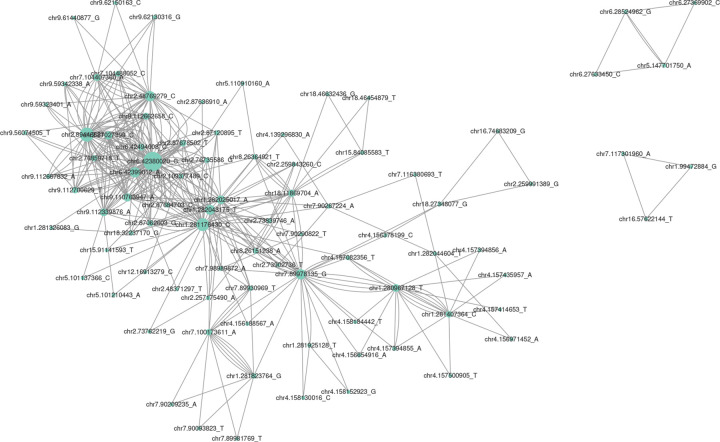
Visualization of epistatic network for XOR triangles with significant 3-way epistatic interactions. Each node represents a SNP, with the size of the node indicating the degree of interaction. The edges between nodes represent the pairwise epistatic interactions with the XOR model. Note that pairs of nodes may be interconnected by multiple edges, stemming from our approach of incorporating edges based on triangular motifs. If an edge involves more than one triangle, then there will be more than one edge connected.

We further investigate the association between triangular motifs in epistatic networks and the presence of higher-order epistasis. To this end, we investigated the third-order epistatic *p*-values of all triangles in the network using the 3-way extension of the methodology presenting in Batista et al. 2023 [12], including 1,033 triangles coded in XOR and 9 triangles coded in Cartesian coordinates. Remarkably, none of the nine triangular motifs in the Cartesian model exhibit significant 3-way epistasis interactions, as determined by an adjusted *p*-values threshold of less than 0.05. Conversely, approximately 13% of XOR triangles (132 out of 1,033) did manifest significant 3-way epistasis (visualized in Figure 4). We further submitted the identical SNP names (N=88) involved in the 132 significant triangles in XOR to g:Profiler (with a 1 Mb range) and identified 12 biological terms (File S7).

### XOR captures additional epistatic interactions that links communities in Cartesian

**Figure 5.**
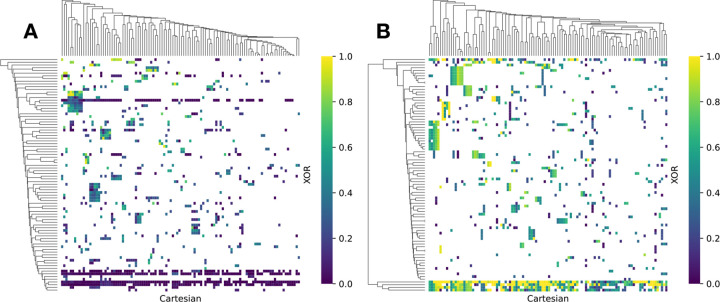
Cluster heat maps comparing the communities from different models based on normalized AUC. The comparison is directional (Section). A) visualization of the comparison from XOR to Cartesian. The comparison from Cartesian to XOR is shown in B).

The comparative assessment of community assignments across both networks renders further insights about the common SNPs. The diagram, depicted in Figure 5, describes the similarities of community assignments based on the normalized AUC defined in Equation 7 in Section.

The results elucidate the efficacy of the XOR model in identifying additional epistatic interactions, thereby enriching our understanding of the genetic architecture by linking communities that are otherwise considered distinct in Cartesian. This is evidenced by the existence of five expansive network communities within the XOR epistatic network, which exhibit shared common SNPs with a majority of communities identified using the Cartesian model. Moreover, this pattern of SNP sharing is not restricted to the largest network communities. Smaller communities also demonstrate a consistent pattern of shared SNPs across both models. Hierarchically clustered heatmaps shown in Figure 5 reinforce this observation, revealing a small fraction of communities that manifest congruent similarity patterns.

### Enrichment analysis

#### Network-scale enrichment analysis

**Figure 6.**
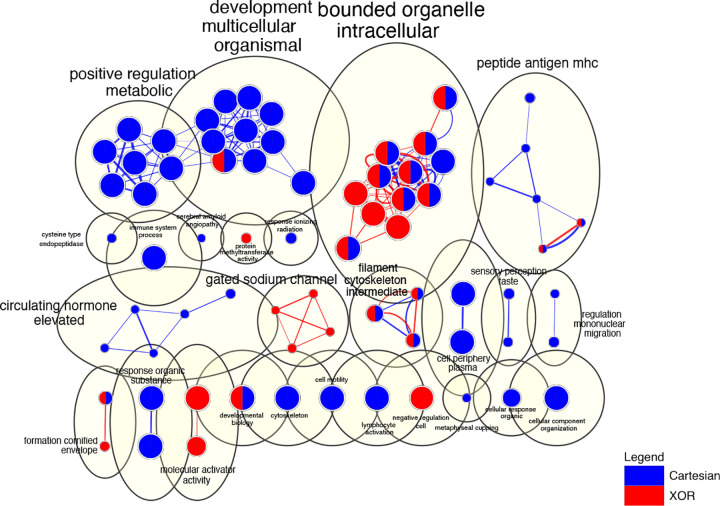
The EnrichmentMap depicts a network of terms extracted from SNPs within different epistatic networks (interaction models). Nodes correspond to biological terms generated from g:Profiler, while edges denote shared genes between terms. AutoAnnotate is employed to aggregate strongly interconnected nodes using cycles, with the theme of the enclosed terms delineated beside each cycle. The node size and edge thickness are proportionate to the gene count they represent. The origin of nodes and edges is indicated by a color coding scheme, where blue denotes origination from Cartesian and red from XOR.

The comparison of interaction models at the network level in *R. norvegicus* using g:Profiler analysis reveals numerous functional categories for the lowest level in the hierarchy. For specific details for each category, please refer to supplementary file S2 and their visualization in Figure 6.

For GO:BP, most of the XOR terms revolve around lectin response and cell-mediated cytotoxicity while Cartesian terms primarily include sensory taste perception, immune responses, developmental processes, and regulation of metabolic process(seen in Figure 6). Networks share the terms for peptide antigen MHC (major histocompatibility complex) (visualized in Figure 6 and refer to the complete table in supplementary file S2). For GO:CC, the XOR network includes terms for sodium-gated channel complex (shown in Figure 6) and the Cartesian network includes polymeric cytoskeletal fiber. The shared terms include MHC protein complex which is one of the clusters shared by XOR and Cartesian in Figure 6. The largest shared cluster between the two interaction models is ”bounded organelle intracellular” which includes all membrane-bound intracellular organelles. For GO:MF, the XOR network includes terms relating to sodium channel activity and methyltransferase activity, while the Cartesian network includes terms for taste receptor activity. Shared terms comprise the MHC protein complex.

For almost all of the categories, the shared terms between Cartesian and XOR networks include immunity and immune response terms. Under the KEGG pathways, the Cartesian network includes terms related to diseases (immune system related), while XOR terms include tuberculosis and signaling pathways related to development. Shared terms between the two include one signaling pathway and diseases. For the REAC category, terms in Cartesian revolve around EGFR and ERBB2 signaling while XOR include terms such as the formation of the cornified envelope and AKT signaling. The two interaction models share terms related to developmental biology and keratinization. For the other sources such as ”Transcription Factor” and ”miRNA”, please refer to supplementary file S2 in sheets TF-CartvsXOR and miRNA-CartvsXOR, respectively. Overall, across all categories, there are 40 terms common to both interaction models, 148 unique terms in Cartesian, and 108 unique terms in XOR.

For the broad categorization (first child of the parent) analysis, almost all categories have shared terms relating to immunity. For specific details for each category, please refer to supplementary file S3. For the Cartesian model, the broad categories mostly include reproductive and metabolic processes, sensory system, signaling, and binding activity. For XOR, the terms comprise mostly of terms relating to signal transduction, cellular component organization, and biological oxidation.

#### Community-specific enrichment analysis for different interaction models

The community-scale biological enrichment analysis unveils the distinct and shared functional roles of each SNP community.

**Figure 7.**
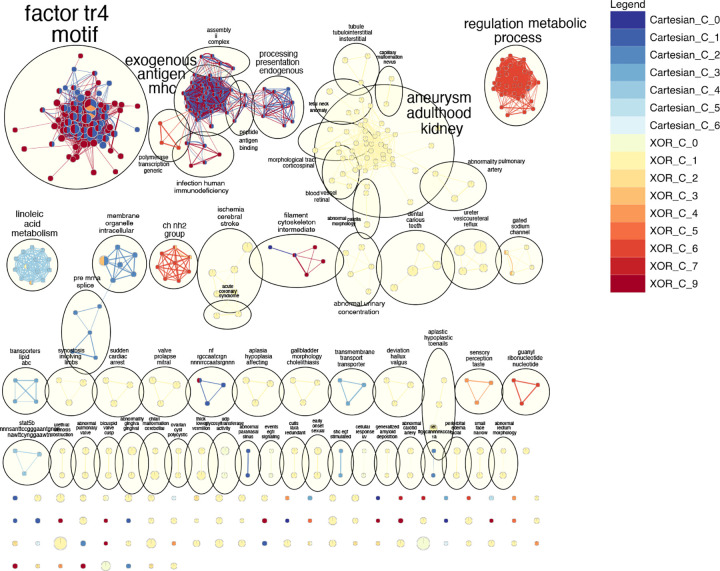
The EnrichmentMap portrays a community-scale comparison of terms derived from SNP communities within distinct epistatic network models. Nodes symbolize biological terms generated from g:Profiler and edges signify the shared genes among these terms. AutoAnnotate clusters closely linked nodes into communities, with each cluster’s overarching theme inscribed beside the cycle. Node size and edge breadth are scaled to the quantity of genes they signify. The origin of nodes and edges is depicted with colors shown in the legend.

The largest term cluster within the EnrichmentMap in Figure 7 is named “factor tr4 motif”, encompassing 92 terms. This cluster is composed of terms from SNP community 1 of the Cartesian model, alongside communities 4 and 9 of the XOR model. The second largest cluster, “aneurysm adulthood kidney”, consists of 40 terms exclusively from SNP community 1 of the XOR model. The third largest cluster is “exogenous antigen mhc” with 37 terms. This includes terms from SNP community 1 of the Cartesian model and communities 6 and 9 of the XOR model. The presence of terms from both models in this cluster suggests a shared significance in the underlying biological processes they represent. The fourth largest cluster, “regulation metabolic process”, includes 32 terms drawn from SNP community 1 of the Cartesian model and SNP community 6 of the XOR model. Major term clusters that are captured by both models include “factor tr4 motif” and “exogenous antigen mhc”. These clusters illustrate the functional similarity between SNP community 1 of the Cartesian model and SNP communities 9 and 6 of the XOR model.

In contrast, some clusters are characterized by a single interaction model. “Aneurysm adulthood kidney” (the second largest term community) and its adjacent communities are primarily enriched with terms from SNP community 1 of the XOR model. Similarly, “regulation metabolic process” is predominantly associated with terms from SNP community 6 of XOR, with a minor contribution (N=1) from SNP community 1 of Cartesian. Another cluster, “linoleic acid metabolism”, mainly consists of terms from SNP community 4 of the Cartesian model, underscoring the unique contributions of each interaction model.

The comparison between interaction models reveals that the XOR model identifies a broader spectrum of biological terms that could be linked to BMI. A similar result was observed in the epistatic analysis our SNPs were derived from [12]. In particular, clusters such as “blood vessel retinal” (N=3), “abnormal urinary concentration” (N=5), “dental carious teeth”, and “abnormal rectum morphology” (N=4) reflect terms related to rat visual and digestive systems.

## Discussion

Our comparative network analysis on epistatic networks reveals that distinct network structures emerge from different network models. The reason for this disparity is rooted in XOR’s improved sensitivity to epistatic interactions compared to the Cartesian model. We found that the XOR model can capture additional epistatic interaction between the same set of SNPs captured by Cartesian and the second-order XOR model presents a unique network motif that can be used to discover higher-order epistasis. Through functional enrichment analysis at the network community scale, we uncovered that the XOR model effectively identifies additional biologically relevant terms and functions, which the Cartesian model fails to detect. Our result not only highlights the capability of XOR in implicating potentially important biological relationships but also highlights the critical role of network structure analysis in comprehending feature interactions. Our findings, therefore, offer new evidence on the significance of the XOR model and the use of network-based investigation in the realm of epistasis analysis for complex traits.

### Distinct epistatic networks evolve under each interaction model

We compare epistatic networks with different interaction models. The compared networks are determined by the threshold, *τ*, that maximizes the network modularity. This ensures that the networks are organized into the best possible network modules.

Figure 2 summarizes the structure discrepancy between the two epistatic networks. The XOR network is characterized by a larger number of nodes and edges compared to the Cartesian network. We further observe that most SNPs in both networks are closely located with each other on the chromosomes (Table 1), and most edges in the Cartesian network are also present in the XOR network, but not the other way around (Table 2). This implies that the XOR model captures more epistatic interactions within a similar set of SNPs.

A significant difference is also observed in the network community structure. The XOR network is dominated by its largest connected component, as additional edges in the XOR network link different components found in the Cartesian model. This leads to a more unified network community in the XOR network, while the Cartesian network is more fragmented.

We also observe that the XOR network contains a greater number of triangles (*n* = 1,033) than Cartesian (Figure 2-E). We consider the triangle motifs in Cartesian (*n* = 9) are likely false-positives for higher-order epistasis as the positions of the corresponding SNPs are adjacent. Although close cis-acting epistasis is another possible explanation, the Cartesian triangles likely arise from high levels of LD. As we test the significance of these triangle motifs for 3-way epistasis, we find that approximately 13% of the triangles in the XOR network have a significant adjusted *p*-value after secondary 3-way analysis. This result suggests that the XOR model, when combining network investigation, can discover higher-order epistatic interactions through the topology of second-order interactions. This finding is encouraging because if higher-order interactions can be discovered using the network structure of lower-order epistasis, the time complexity of discovering higher-order interactions will be greatly reduced.

Last but not least, the XOR network has a lower modularity compared to the Cartesian network. This lower modularity in XOR can be attributed mainly to the additional epistatic interactions it captures, which tend to bridge distinct network communities found by the Cartesian model. These bridging edges often span across different network communities, thereby reducing modularity. Additionally, the presence of triangles in the XOR network, especially those that cross community boundaries, further contributes to the reduced modularity. In contrast, the Cartesian network’s near absence of triangles and higher number of connected network components result in higher modularity, reflecting its more segregated community structure.

### Network level enrichment analysis reveals shared and unique biological signals between interaction models

Our enrichment results at the network level indicate both shared and unique biological signals in each model’s network. GO terms and KEGG pathways reveal that immunity-based enrichments are shared between Cartesian and XOR models at both high and low levels of biological organization that we assessed in our enrichment analysis (see supplementary files S2 and S3). This is somewhat expected due to ubiquity of immunity-based enrichments regularly observed across systems and phenotypes [56–60] including in the results of the original epistasis analysis that this work is inspired by [12]. Immune functions are underlain by diverse gene networks that are integral to general stress responses observed in many systems [43,57,61]. High BMI likely induces an array of stress response mechanisms which, in turn, activate genes and gene networks related to immunity. Our results suggest that, regardless of epistatic model used, immunity-related signals are detectable and prevalent in our networks. Additionally, REAC results indicate that keratinization is a process shared in Cartesian and XOR networks at both levels of enrichment analysis (see supplementary files S3). It has been observed in humans that morbid obesity can lead to several skin disorders involving irregular keratinization [62,63]. In rats, it is unclear if these enrichments point to processes analogous to those observed in humans. Additional experiments are required to elucidate this potential similarity between humans and rats.

Specific to the Cartesian network, we observe biological processes, molecular functions, and KEGG pathways involved in taste reception (see supplementary file S3). These terms have a clear connection to ingestion and metabolism [64,65] and are thus likely directly related to BMI. In the XOR network, we observe biological processes, molecular functions, and KEGG pathways associated with cellular organization, biogenesis, and cell-cell signaling (see supplementary file S3). These are accompanied by enrichments associated with lectin receptor activity. However, these terms are involved in immunity and thus are likely part of the shared immunity-related terms observed in the Cartesian network as well. Interestingly, also in XOR, we observe cellular component enrichments in terms associated with ion channels (see supplementary file S3). The roles of ion channels in the development of obesity in rats have been well documented and are involved in adipose cell proliferation, food intake, and gastric emptying (overeating) [66]. In REAC, Cartesian and XOR networks are both enriched for cell-cell signaling pathways but the pathways are distinct in that Cartesian is enriched for EGFR and ERBB signaling while XOR is enriched for AKT signaling (see supplementary file S3). Finally, our enrichment analysis of SNPs involved in significant 3-way XOR interactions, derived from the triangle structures (Figure 4), yields 12 terms. Most of these terms are associated with gene regulation as the bulk of the enrichments are associated with transcription factors (see supplementary file S7). However, one term, “beta-galactosidase activity” likely has links to metabolism and/or BMI. Indeed, in a human study, senescence-activated beta-galactosidase activity has been linked to altered glucose metabolism and body fat distribution [67]. Taken together, our network level enrichment results indicate that broad biological systems can be implicated using either interaction model in regards to BMI in rats. Similar results were observed in the original epistatic analysis where terms shared by both interaction models were involved in immunity [12]. However, each model has the potential to highlight unique pathways that would have been missed if only one interaction model was utilized in this system. Moreover, our enrichment results align with findings from our network analysis in that each epistatic network’s unique topology likely highlights distinct genetic architectures associated with the phenotype. Our analysis here serves as evidence illustrating that the Cartesian interaction model (or any one model) alone is not adequate to explore all of the possible epistatic interactions that occur in living systems and should be supplemented by other models/penetrance functions, including non-linearly separable models, like XOR.

### Community level enrichment analysis highlights the advantages of the XOR model and network investigation

The investigation of epistatic networks through community-based enrichment analysis demonstrates that the XOR model identifies network structures with potential biological relevance better than Cartesian in this system and phenotype. Our community-level enrichment analysis is illustrated in Figure 7, which suggests that although the Cartesian model identifies a certain amount of major term clusters, the XOR model identifies more (Files S5 and S6). The resulting terms from the XOR model not only are involved with all major clusters from Cartesian but also uncover additional clusters of BMI-related terms. For example, community 1 of the XOR model uniquely reveals the “aneurysm adulthood kidney” term cluster, a discovery not observed using the Cartesian model (File S5). This community includes terms associated with ion channel activity and S-methyltransferase activity both of which have been shown to have links to obesity in rats and mice [66,68]. Furthermore, community 6 “regulation metabolic process” is chiefly built by terms identified by the XOR model (all aside from one term; Files S5 and S6). This community is made up of primarily anabolic and biosynthetic biological processes (File S5). This underscores XOR’s enhanced capability in revealing relevant functions that Cartesian can not. Specific to the terms, we consider that XOR identifies additional biological terms related to the visual and digestive systems of rats. The novel terms identified in Community 1, such as ”Nystagmus-induced head nodding,” ”Down-sloping shoulders,” ”Blue irides,” etc., primarily describe anatomical characteristics. These terms could be associated with BMI and obesity through growth and development, but further research is required to strengthen this claim. We consider such a deeper and more comprehensive understanding can be attributed to the ability of XOR to integrate additional genetic interactions that subsume multiple network modules of Cartesian into one. The results from the community-level enrichment mirror those from the original analysis in which XOR-specific terms were enriched for biological functions and processes associated with metabolism [12].

In conclusion, our community-level enrichment analysis clearly highlights the efficacy of XOR as a model of epistasis in this system and phenotype. The unique interactions and biological insights identified by XOR at the network level and via GSEA are pivotal in revealing a more profound understanding of the genetic architecture of BMI in rats. These results also highlight the need for network structure analysis and the unique advantages of XOR coding, and perhaps other interaction models, in epistasis studies.

### Future work

The construction of the epistatic networks leverages the precise chromosomal positions of SNP to discriminate individual nodes. Consequently, nodes and edges in close proximity are identified as separate entities. However, this approach introduces redundancy due to the presence of analogous nodes and edges, which complicates the network’s analysis. There is a clear need for advanced analytical methods that can account for the similarities among nodes and edges. To overcome this challenge, the adoption of network representation learning methods, especially graph neural networks [69], emerges as a promising solution. These techniques excel at generating embedding representations for the information associated with the nodes [70,71]. In the context of our epistatic networks, these embeddings are capable of identifying nodes and edges with similar chromosomal positions, thereby substantially improving the accuracy and efficiency of network analysis.

## Conclusions

This comparative network analysis illustrates that the use of multiple interaction models allows for the elucidation of complex epistatic interactions in a model system. Although both interaction models identify respective network structures, the XOR model facilitates the integration of distinct network communities in Cartesian, thereby unveiling novel biological functions via community-scale enrichment analysis. Specifically, the first community within the XOR network has revealed several HP terms associated with the rat visual and digestive systems that had not been previously implicated to be involved with BMI, obesity, or metabolism. Furthermore, the XOR model’s propensity to identify unique triangular motifs, with approximately 13% indicative of three-way statistical epistasis, illustrates a novel approach for identifying complex interactions through the topology of lower-order interactions. This approach can be used to simplify the identification of high-order interactions in high-dimensional data, which is often computationally expensive.

To summarize, this comparative analysis underlines the importance of network analysis in epistasis studies. A network brings together different entities through their interactions, enabling a holistic view over a vast number of complex intertwined interactions. Using this approach, we identify novel biological insights and evidence of higher-order epistasis.

## Figures and Tables

**Figure 3.**
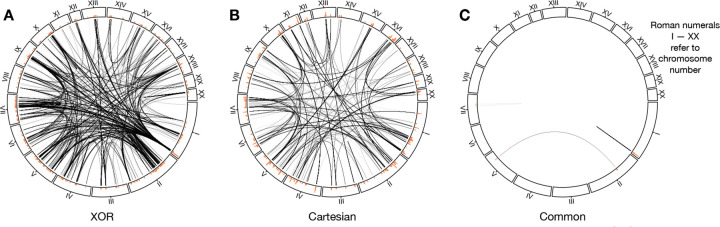
Visualization of High-Modularity epistatic networks. (A) For XOR, *τ* = 0.0434 yields a network comprising 10,736 edges and 2,803 nodes. (B) For Cartesian, *τ* = 0.0488 results in a network with 3,152 edges and 1,579 nodes. (C) The two networks have an overlap of 99 edges and 416 nodes

**Table 1.** Comparison of SNPs in epistatic networks with different interaction models

Range	XOR in Cartesian		Cartesian in XOR	
	Quantity	Percentage	Quantity	Percentage
0 Mb	416	14.84%	416	26.35%
1 Mb	1,979	70.60%	1,317	83.41%
2 Mb	2,209	78.81%	1,380	87.40%
3 Mb	2,354	83.98%	1,420	89.93%
4 Mb	2,416	86.19%	1,485	94.05%
5 Mb	2,467	88.01%	1,527	96.71%
6 Mb	2,530	90.26%	1,540	97.53%
7 Mb	2,559	91.30%	1,540	97.53%
8 Mb	2,571	91.72%	1,543	97.72%
9 Mb	2,614	93.26%	1,546	97.91%
10 Mb	2,656	94.76%	1,546	97.91%
All	2,803	100.00%	1,579	100.00%

**Table 2.** Comparison of edges in epistatic networks with different interaction models

Range	XOR in Cartesian		Cartesian in XOR	
	Quantity	Percentage	Quantity	Percentage
0 Mb	99	0.92%	99	3.14%
1 Mb	315	2.93%	682	21.64%
2 Mb	371	3.46%	834	26.46%
3 Mb	452	4.21%	1,070	33.95%
4 Mb	477	4.44%	1,217	38.61%
5 Mb	495	4.61%	1,261	40.01%
6 Mb	605	5.64%	1,302	41.31%
7 Mb	774	7.21%	1,337	42.42%
8 Mb	811	7.55%	1,691	53.65%
9 Mb	840	7.82%	2,014	63.90%
10 Mb	840	7.82%	2,312	73.35%
All	10,736	100.00%	3,152	100.00%

## Data Availability

Rat phenotype data and GWAS summary statistics are available at https://library.ucsd.edu/dc/object/bb83725195. Rat genotype data are available at https://library.ucsd.edu/dc/object/bb15123938. The implementations of the algorithms for 2-way and 3-way epistasis detection given in Python are offered via GitHub at https://github.com/EpistasisLab/epistasis detection. The implementations of the network investigation given in Python are offered via GitHub at https://github.com/shazhendong/Network Epistasis. The scripts to perform GSEA and obtain the parents of interest and genes given a set of SNPs are available on the Open Science Framework (OSF): https://osf.io/qfnec/.

## Abbreviations

AKT: AK strain Transforming
AUC: Area Under the Curve
BMI: Body Mass Index
BP: Biological Process
CC: Cellular Component
EGFR: Estimated Glomerular Filtration Rate
ERBB2: ERythroBlastic oncogene B receptor tyrosine kinase 2
FDR: False Discovery Rate
GO: Gene Ontology
GSEA: Gene Set Enrichment Analysis
GWAS: Genome-Wide Association Study
KEGG: Kyoto Encyclopedia of Genes and Genomes
LD: Linkage Disequilibrium
Mb: MegaBase(s) (1e6 base pairs)
MHC: Major Histocompatibility Complex
MF: Molecular Function
OSA: Over-Representation Analysis
QTL: Quantitative Trait Locus
REAC: REACtome pathway database
SNP: Single Nucleotide Polymorphism
TFs: Transcription Factors
XOR: eXclusive OR
